# The Northampton General Infirmary

**Published:** 1901-06-15

**Authors:** 


### The Institutional Workshop.

### THE NORTHAMPTON GENERAL INFIRMARY.

It is a serious point in the history of any hospital when its managers discover, as the managers of the Northampton General Infirmary lately discovered, that by mere lapse of time, and perhaps by the fact that their own success has made them indifferent to what has gone on in other places, they have laid themselves open to the charge of being out of date, and of being behindhand in their sanitary standard. Lucky is the hospital, however, which, when it finds itself in this position, is able, as the Northampton Infirmary has been, to fall back upon its resources, and by realising some of its assets to rejuvenate its buildings and to place itself again on a level with its compeers. Some little time ago the weekly board of the Northampton General Infirmary began to feel that the institution for the management of which they were responsible was in many respects in an unsatisfactory condition, and in consequence of this feeling a deputation was appointed to visit Guy's Hospital with the object of investigating the manner in which things were done in a thoroughly up-to-date institution, and of thus arriving at some standard of comparison by which to judge as to the changes which ought to be made in their own hospital. This deputation of three, headed by Earl Spencer, visited Guy's Hospital on April 24th, and its members were evidently much impressed with what they saw, and especially with the contrast offered by the conditions of the wards, the laundries, and the nursing of this great hospital with what they were accustomed to in Northampton, and they at once recommended that a series of alterations should be made in their own infirmary, especially in regard to the flooring in the wards and the arrangements of the ward kitchens and lavatories. They also advised that the bedding and ward furniture should be entirely renewed. In their recommendations, however, this deputation hardly had the full courage of their convictions, for instead of advising that the improvements should be at once put in hand they advised that the change should be undertaken little by little, beginning with one ward and gradually working through the building. It was evidently felt, however, by some that this was hardly a sufficiently complete and thorough mode of dealing with so serious a condition of affairs as had been shown to exist, and the joint committee, which was appointed by the Quarterly Court to carry out the alterations, requested Sir Henry Burdett, K.C.B., to give his advice in the matter. This he consented to do, and after inspecting the building he sent in a report to the governors, advising that very extensive alterations and repairs should be forthwith undertaken. He pointed out that although externally a renovation of the building had been carried out, the condition internally was very unsatisfactory. The walls, many of the floors, the ceilings, the ceiling ventilation, the arrangements of the bathrooms and water-closets, most of the furniture, the whole of the sanitary, lavatory, scullery, and bath fittings, he said, required renewal, and from the nature of his comments we can hardly wonder at the thoroughgoing character of his advice—namely, that the state of affairs could only be remedied by stripping the wards, and renewing everything which was out of date, insanitary, or unsuited to the treatment of disease under modern conditions. In regard to the nursing, he very properly speaks of the importance of good accommodation being provided for an efficient nursing staff under the most approved hygienic conditions. At the present time it appears that the nursing staff at the infirmary is housed in rooms, some of which are connected with the wards, while some are in juxtaposition with the sculleries, &c.; and Sir Henry Burdett says that there can be no doubt that the time has arrived when it is essential that the governors should provide adequate accommodation for their nurses by the erection of a nurses' home, by prefer ence in the grounds, where he thinks that an excelent site could be found for the purpose.

We need not here go further into the deficiencies of which Sir Henry Burdett speaks in his report. It is of more interest to point out what he says in regard to the financial side of the question, the side which in most hospitals is the bugbear which stands in the way of all improvement. Without going into any detailed estimate he holds that the requisite alterations, including the cost of a new nurses' home, could probably be done for a sum of between £15,000 and £20,000, and, meeting the objection which he felt was sure to crop up to the effect that it would be impossible to raise such a sum, he pointed to the example of various of the London hospitals, where hundreds of thousands of pounds had been expended within the last few years to renew old buildings and make them hygienically satisfactory. His strong point, however, was that the Northampton General Infirmary really has the money in hand already. It has invested funds the capital value of which amounts to upwards of £60,000. Now, as Sir Henry Burdett points out, the annual expenditure seems in round numbers to be about £7,600; and as it is held by the highest authorities that a reserve fund equal to five years' annual expenditure, or in the case of N orthampton say £40,000, constitutes a reserve adequate to protect a hospital from any financial risk which it is likely to have to face, it is clear that the good people at North ampton have in hand at least £20,000 which they can properly expend upon the renovation of the infirmary, the nursing home, wards, &c., providing it is agreed to make an immediate and continuous effort to raise a renewal fund to replace the capital so expended. A point on which Sir Henry Burdett laid considerable stress was that it would not be wise to renovate only a single ward as a beginning, but that all the wards in one wing should be undertaken together. At a meeting of the special committee appointed to consider this report, held on May 29th, it was resolved that it is desirable that the recommendations of the deputation, as supplemented by those of Sir Henry Burdett, be at once carried out in the children's ward, and be extended to the whole of the wing in which that ward is situated; that an appeal be made to the public for support for this large scheme; and that the Finance and House Committee be empowered to borrow provisionally a sum not exceeding £15,000 from the capital fund of the institution to secure that the work shall be properly carried out.

We understand that the Special Court of Governors, held on June 8th, adopted this resolution.

